# Supplementary material for: Is there a “sweet spot” of model complexity for qualitative models used in Ecosystem-Based Management?
Source: PLoS One. 2025 Jul 17;20(7):e0328505. doi: 10.1371/journal.pone.0328505 (PMC12270125; doi:10.1371/journal.pone.0328505)
Supplement: S1 File — Table SA1. Aggregate groupings from larger WSS63 model to smaller WSS28. Table SA2. Biomass and pedigrees for the WSS28 Rpath model including pedigree for diets (SA3). Table S3. Diet matrix for the Rpath WSS28 model. (DOCX) [file pone.0328505.s001.docx]

# Supplemental Material

Table SA1. Aggregate groupings from larger WSS63 model to smaller WSS28.

| **WSS Ecopath Model**  (62 Functional Groups + Fishery) | **Rpath and QNM Models**  (27 Functional Groups + Fishery) |
| --- | --- |
| Whales | Whales |
| Toothed cetaceans | Toothed cetaceans |
| Seals | Seals |
| Sea birds | Sea birds |
| Sharks | Sharks |
| Large pelagics | Large pelagics |
| Cod (4 Stanzas) | Demersal Piscivores |
| Silver hake (3 stanzas) |  |
| Halibut (3 stanzas) |  |
| Pollock (2 stanzas) |  |
| Demersal piscivores (2 stanzas) |  |
| Large benthivores (2 stanzas) | L Benthivores |
| Skates (2 stanzas) |  |
| Dogfish |  |
| Redfish (2 stanzas) |  |
| American plaice (2 stanzas) |  |
| Flounders (2 stanzas) |  |
| Haddock (2 stanzas) |  |
| Longhorn sculpin (2 stanzas) |  |
| Small-medium benthivores |  |
| Atlantic Herring (2 stanzas) | Small pelagics |
| Other pelagic |  |
| Mackerel |  |
| Mesopelagic | Mesopelagics |
| Squids | Squids |
| Shrimps | Shrimp |
| Scallop | Bivalves |
| Bivalves |  |
| Small crabs | Small crab other arthropoda |
| Other arthropoda |  |
| Other molluscs | Other molluscs |
| Lobster | Megabenthos |
| Large crabs |  |
| Echinoderms |  |
| Sessile benthic groups |  |
| Worms | Worms |
| Meiofauna | Meiofauna |
| Gelatinous zooplankton | Gelatinous Zooplankton |
| Macrozooplankton | Macrozooplankton |
| Mesozooplankton | Mesozooplankton |
| Microzooplankton | Microzooplankton |
| Microflora | Microflora |
| Phytoplankton | Phytoplankton |
| Discards | Discards |
| Detritus | Detritus |

Table SA2. Biomass and pedigrees for the WSS28 Rpath model including pedigree for diets (SA3).

| Group | Biomass | PB | QB | Fishery | Pedigree (biomass) | Pedigree (PB) | Pedigree (QB) | Pedigree (fishery) | Pedigree (diet) |
| --- | --- | --- | --- | --- | --- | --- | --- | --- | --- |
| Whales | 0.407075 | 0.071 | 4.94 | 0 | 0.3 | 0.5 | 0.5 | 0.2 | 0.5 |
| Toothed cetaceans | 0.049754 | 0.18 | 14.5 | 0 | 0.3 | 0.5 | 0.5 | 0.2 | 0.5 |
| Seals | 0.04383 | 0.147007 | 7.338632 | 0 | 0.6 | 0.3 | 0.3 | 0.2 | 0.5 |
| Sea birds | 0.00617 | 0.25 | 87.6 | 0 | 0.5 | 0.5 | 0.5 | 0.2 | 0.5 |
| Sharks | 0.026025 | 0.18 | 4.78 | 0.002091 | 0.3 | 0.4 | 0.4 | 0.5 | 0.3 |
| Large pelagics | 0.023573 | 0.4 | 4.24 | 0.005068 | 0.5 | 0.3 | 0.4 | 0.3 | 0.5 |
| D piscivores | 4.082286 | 0.534111 | 2.868397 | 0.325171 | 0.3 | 0.3 | 0.3 | 0.3 | 0.5 |
| L benthivores | 4.240253 | 0.379831 | 3.254083 | 0.182276 | 0.3 | 0.3 | 0.3 | 0.3 | 0.5 |
| Skates | 0.137757 | 0.232082 | 2.447539 | 0.000799 | 0.3 | 0.3 | 0.3 | 0.3 | 0.5 |
| Small pelagics | 6.294171 | 0.695925 | 3.475426 | 0.999061 | 0.5 | 0.5 | 0.3 | 0.3 | 0.5 |
| Other pelagic mesopelagic | 0.878285 | 0.743277 | 3.532147 | 0.019708 | 0.8 | 0.7 | 0.7 | 0.2 | 0.7 |
| Squids | 0.173893 | 4 | 11.33333 | 0.001637 | 0.7 | 0.5 | 0.5 | 0.2 | 0.7 |
| Megabenthos | 0.458598 | 0.82219 | 5.481265 | 0.220424 | 0.6 | 0.6 | 0.8 | 0.1 | 0.7 |
| Small crab other arthropoda | 1.818481 | 2.759435 | 18.39623 | 0 | 0.6 | 0.5 | 0.8 | 0.2 | 0.7 |
| Shrimps | 1.302563 | 3 | NA | 0.000162 | 0.8 | 0.5 | 0.8 | 0.1 | 0.5 |
| Bivalves | 64.01978 | 0.69 | NA | 0.117151 | 0.6 | 0.5 | 0.8 | 0.1 | 0.7 |
| Other molluscs | 2 | 0.75 | NA | 0.00236 | 0.6 | 0.5 | 0.8 | 0.2 | 0.7 |
| Worms | 7.345485 | 1.25 | NA | 0 | 0.6 | 0.5 | 0.8 | 0.2 | 0.7 |
| Meiofauna | 42.75382 | 1.32583 | 8.83887 | 0.03026 | 0.6 | 0.5 | 0.8 | 0.2 | 0.7 |
| Gelatinous zoop | 0.5204 | 15.51 | 62.05 | 0 | 0.5 | 0.6 | 0.6 | 0.2 | 0.6 |
| Macrozoop | 41.31 | 3.04 | 19.5 | 0 | 0.3 | 0.5 | 0.5 | 0.2 | 0.6 |
| Mesozoop | 23.79555 | 29.2 | 73 | 0 | 0.3 | 0.5 | 0.5 | 0.2 | 0.6 |
| Microzoop | 5.800508 | 82.8 | NA | 0 | 0.8 | 0.6 | 0.6 | 0.2 | 0.6 |
| Microflora | 3.678861 | 104.938 | NA | 0 | 0.8 | 0 | 0 | 0.2 | 0 |
| Phytoplankton | 33.664 | 70.639 | NA | 0 | 0.5 | 0 | 0 | 0.2 | 0 |
| Discards | 0.063293 | NA | NA | 0 | 0.8 | 0 | 0 | 0.2 | 0 |
| Detritus | 1 | NA | NA | 0 | 0.8 | 0 | 0 | 0.2 | 0 |
| Fishery | NA | NA | NA | NA | 0 | 0 | 0 | 0 | 0 |

Table S3. Diet matrix for the Rpath WSS28 model.

| Group | Whales | Toothed cetaceans | Seals | Sea birds | Sharks | Large pelagics | D piscivores | L benthivores | Skates | Small pelagics | Other pelagic mesopelagic |
| --- | --- | --- | --- | --- | --- | --- | --- | --- | --- | --- | --- |
| Whales | 0 | 0 | 0 | 0 | 0 | 0 | 0 | 0 | 0 | 0 | 0 |
| Toothed cetaceans | 0 | 0 | 0 | 0 | 0 | 0 | 0 | 0 | 0 | 0 | 0 |
| Seals | 0 | 0 | 0 | 0 | 0.00651199 | 0 | 0 | 0 | 0 | 0 | 0 |
| Sea birds | 0 | 0 | 0 | 0.00266399 | 0 | 0 | 0 | 0 | 0 | 0 | 0 |
| Sharks | 0 | 0 | 0 | 0 | 0 | 0 | 0 | 0 | 0 | 0 | 0 |
| Large pelagics | 0 | 0 | 0 | 0 | 0 | 0.00222 | 0 | 0 | 0 | 0 | 0 |
| D piscivores | 0.004802 | 0.2437588 | 0.1276689 | 0.000721 | 0.1848318 | 0.06917194 | 0.05424953 | 0.00554105 | 0.00308048 | 0 | 0 |
| L benthivores | 0.012545 | 0.09793591 | 0.2855617 | 0.00507699 | 0.3280216 | 0.1511799 | 0.04674805 | 0.00413294 | 0.05460171 | 0 | 0 |
| Skates | 0 | 0 | 0.00643199 | 0 | 0.000808 | 0.002839 | 0.00049652 | 0 | 0 | 0 | 0 |
| Small pelagics | 0.173918 | 0.3776346 | 0.5207465 | 0.3529089 | 0.4223085 | 0.6149244 | 0.1726697 | 0.00143368 | 0.02304704 | 0 | 0 |
| Other pelagic mesopelagic | 0.0194 | 0.00739699 | 0.05680194 | 0.07901775 | 0.04387695 | 0.1276559 | 0.02289399 | 0.01478033 | 0.03303853 | 0 | 0 |
| Squids | 0.011769 | 0.2679567 | 0.002057 | 0.03595289 | 0.01354798 | 0.01555398 | 0.02838177 | 0.00181741 | 0.00600834 | 0 | 0 |
| Megabenthos | 0 | 0 | 2.10E-05 | 0 | 9.30E-05 | 7.30E-05 | 0.00377164 | 0.00180497 | 0.00659312 | 0 | 0 |
| Small crab other arthropoda | 0 | 0 | 0 | 0 | 0 | 0 | 0.03455577 | 0.07374978 | 0.1577646 | 0.02415398 | 0.060237 |
| Shrimps | 0 | 0 | 0.000711 | 0 | 0 | 0.00331 | 0.07398107 | 0.08974432 | 0.07314944 | 0.0622156 | 0.03105934 |
| Bivalves | 0 | 0 | 0 | 0 | 0 | 0 | 0.00158274 | 0.03253376 | 0.00083476 | 0 | 0 |
| Other molluscs | 0 | 0 | 0 | 0 | 0 | 0 | 0.00104459 | 0.00598785 | 0.00203087 | 0 | 0 |
| Worms | 0 | 0 | 0 | 0 | 0 | 0 | 0.00229067 | 0.09854721 | 0.1947723 | 0.0010416 | 0.0008129 |
| Meiofauna | 0 | 0 | 0 | 0 | 0 | 0.01216899 | 0.00460342 | 0.09481322 | 0.00126187 | 0.00344101 | 0 |
| Gelatinous zoop | 0 | 0 | 0 | 0 | 0 | 5.90E-05 | 0.08581191 | 0.00871915 | 0.00167152 | 0.00017192 | 0.00121196 |
| Macrozoop | 0.726929 | 0 | 0 | 0.315373 | 0 | 0 | 0.4453562 | 0.5160776 | 0.3443648 | 0.8572081 | 0.3833565 |
| Mesozoop | 0.050637 | 0 | 0 | 0 | 0 | 0 | 0.01242108 | 0.0372947 | 0.09778056 | 0.05176783 | 0.5141716 |
| Microzoop | 0 | 0 | 0 | 0 | 0 | 0 | 0 | 0 | 0 | 0 | 0 |
| Microflora | 0 | 0 | 0 | 0 | 0 | 0 | 0 | 0 | 0 | 0 | 0 |
| Phytoplankton | 0 | 0 | 0 | 0 | 0 | 0 | 0 | 0 | 0 | 0 | 0.0024267 |
| Discards | 0 | 0.005317 | 0 | 0.000553 | 0 | 0 | 0 | 0 | 0 | 0 | 0 |
| Detritus | 0 | 0 | 0 | 0 | 0 | 0 | 0 | 0 | 0 | 0 | 0 |
| Import | 0 | 0 | 0 | 0.2077324 | 0 | 0.000844 | 0.00914136 | 0.01302205 | 0 | 0 | 0.006724 |

Table S3. Diet matrix for the Rpath WSS28 model cont.

| Group | Squids | Megabenthos | Small crab other arthropoda | Shrimps | Bivalves | Other molluscs | Worms | Meiofauna | Gelatinous zoop | Macrozoop |
| --- | --- | --- | --- | --- | --- | --- | --- | --- | --- | --- |
| Whales | 0 | 0 | 0 | 0 | 0 | 0 | 0 | 0 | 0 | 0 |
| Toothed cetaceans | 0 | 0 | 0 | 0 | 0 | 0 | 0 | 0 | 0 | 0 |
| Seals | 0 | 0 | 0 | 0 | 0 | 0 | 0 | 0 | 0 | 0 |
| Sea birds | 0 | 0 | 0 | 0 | 0 | 0 | 0 | 0 | 0 | 0 |
| Sharks | 0 | 0 | 0 | 0 | 0 | 0 | 0 | 0 | 0 | 0 |
| Large pelagics | 0 | 0 | 0 | 0 | 0 | 0 | 0 | 0 | 0 | 0 |
| D piscivores | 0.003657 | 0 | 0 | 0 | 0 | 0 | 0 | 0 | 0 | 0 |
| L benthivores | 0.007678 | 0.00109202 | 0 | 0 | 0 | 0 | 0 | 0 | 0 | 0 |
| Skates | 0.000187 | 0 | 0 | 0 | 0 | 0 | 0 | 0 | 0 | 0 |
| Small pelagics | 0.015327 | 0 | 0 | 0 | 0 | 0 | 0 | 0 | 0 | 0 |
| Other pelagic mesopelagic | 0.004734 | 0 | 0 | 0 | 0 | 0 | 0 | 0 | 0 | 0 |
| Squids | 0.037929 | 0.00228014 | 0 | 0 | 0 | 0 | 0 | 0 | 0 | 0 |
| Megabenthos | 0 | 0.00705279 | 0 | 0 | 0 | 0 | 0 | 0 | 0 | 0 |
| Small crab other arthropoda | 0.014737 | 0.09158555 | 0.00969031 | 0.004533 | 0 | 0.03769 | 0.02 | 0.00018408 | 0 | 0 |
| Shrimps | 0.010014 | 0.00126764 | 0 | 0 | 0 | 0.020851 | 0 | 0 | 0 | 0 |
| Bivalves | 0 | 0.3312395 | 0.01410418 | 0 | 0 | 0.332281 | 0 | 0.00118479 | 0 | 0 |
| Other molluscs | 0 | 0.0419751 | 0.01410418 | 0 | 0 | 0.027689 | 0 | 0.00061719 | 0 | 0 |
| Worms | 0.013273 | 0.1380028 | 0.02820811 | 0.015272 | 0 | 0 | 0.070001 | 0.00061719 | 0 | 0 |
| Meiofauna | 0 | 0.281883 | 0.08955499 | 0 | 0 | 0.304589 | 0.070001 | 0.03959263 | 0 | 0 |
| Gelatinous zoop | 0.001571 | 0 | 0 | 0 | 0 | 0 | 0 | 0 | 0 | 0 |
| Macrozoop | 0.890893 | 0 | 0 | 0.123605 | 0 | 0 | 0 | 0.00577469 | 0.161448 | 0.055941 |
| Mesozoop | 0 | 0 | 0 | 0.244326 | 0 | 0 | 0 | 0.00528442 | 0.696038 | 0.47728 |
| Microzoop | 0 | 0 | 0 | 0 | 0 | 0 | 0 | 0.00386473 | 0.095929 | 0.11221 |
| Microflora | 0 | 0 | 0.00685499 | 0.008578 | 0.095 | 0 | 0 | 0.0102941 | 0.005032 | 0.015172 |
| Phytoplankton | 0 | 0 | 0 | 0.069225 | 0.882656 | 0 | 0 | 0.08967555 | 0.041553 | 0.127274 |
| Discards | 0 | 0.00252534 | 0.00134418 | 0 | 0 | 0 | 0 | 0 | 0 | 0 |
| Detritus | 0 | 0.06347585 | 0.8361391 | 0.534461 | 0.022344 | 0.2769 | 0.839998 | 0.8429105 | 0 | 0.212123 |
| Import | 0 | 0.03762027 | 0 | 0 | 0 | 0 | 0 | 0 | 0 | 0 |

Table S3. Diet matrix for the Rpath WSS28 model cont.

| Group | Mesozoop | Microzoop | Microflora | Phytoplankton |
| --- | --- | --- | --- | --- |
| Whales | 0 | 0 | 0 | 0 |
| Toothed cetaceans | 0 | 0 | 0 | 0 |
| Seals | 0 | 0 | 0 | 0 |
| Sea birds | 0 | 0 | 0 | 0 |
| Sharks | 0 | 0 | 0 | 0 |
| Large pelagics | 0 | 0 | 0 | 0 |
| D piscivores | 0 | 0 | 0 | 0 |
| L benthivores | 0 | 0 | 0 | 0 |
| Skates | 0 | 0 | 0 | 0 |
| Small pelagics | 0 | 0 | 0 | 0 |
| Other pelagic mesopelagic | 0 | 0 | 0 | 0 |
| Squids | 0 | 0 | 0 | 0 |
| Megabenthos | 0 | 0 | 0 | 0 |
| Small crab other arthropoda | 0 | 0 | 0 | 0 |
| Shrimps | 0 | 0 | 0 | 0 |
| Bivalves | 0 | 0 | 0 | 0 |
| Other molluscs | 0 | 0 | 0 | 0 |
| Worms | 0 | 0 | 0 | 0 |
| Meiofauna | 0 | 0 | 0 | 0 |
| Gelatinous zoop | 0 | 0 | 0 | 0 |
| Macrozoop | 0 | 0 | 0 | 0 |
| Mesozoop | 0.058572 | 0 | 0 | 0 |
| Microzoop | 0.161911 | 0.099731 | 0 | 0 |
| Microflora | 0.076645 | 0.202492 | 0 | 0 |
| Phytoplankton | 0.702872 | 0.209335 | 0 | 0 |
| Discards | 0 | 0 | 0 | 0 |
| Detritus | 0 | 0.488442 | 1 | 0 |
| Import | 0 | 0 | 0 | 0 |

S4.

The details of results are included in the public github: [RpathQNM/WSS28model/data neutrals at main · NOAA-EDAB/RpathQNM](https://github.com/NOAA-EDAB/RpathQNM/tree/main/WSS28model)
