# Supplementary material for: Is there a “sweet spot” of model complexity for qualitative models used in Ecosystem-Based Management?
Source: PLoS One. 2025 Jul 17;20(7):e0328505. doi: 10.1371/journal.pone.0328505 (PMC12270125; doi:10.1371/journal.pone.0328505)
Supplement: Attachment — Submitted filename: PONE-D-25-18645 Response to Reviewer.docx [file pone.0328505.s004.docx]

**Fisheries and Oceans Canada**	**Jun 23, 2025**

**Bedford Institute of Oceanography**

1 Challenger Drive,

Dartmouth, Nova Scotia

CANADA B3B 1A6

**Athanassios C. Tsikliras**
Academic Editor 
PLOS ONE 

**To Athanassios C. Tsikliras,**

Please find our enclosed revised Research Article for PLOS ONE, “Is there a “sweet spot” of model complexity for qualitative models used in Ecosystem-Based Management?”, by Tam et al., for submission [PONE-D-25-18645]. Alongside this response to reviewers, is:

- A marked up copy of the MS that highlights changes to the original version.
- An unmarked version as the revised “Manuscript”.

Thank you for the time and effort to edit and review this paper. The comments have made it much stronger.

We confirm that this manuscript has not been published elsewhere, nor is it under consideration by another journal. All authors have approved the manuscript and agree with the submission to PLOS ONE.

Sincerely,

Jamie C. Tam, PhD

### Response to Reviewer #1 (Ioannis Keramidas)

Thank you for your thorough and constructive review. We appreciate your thoughtful assessment of this manuscript and we were happy that you agreed with the need for practical research to support the operationalization of ecosystem models into decision making. Please see the responses to your questions below and have included text clearly labeled in the marked version of the MS.

It would be useful to justify the thresholds for removing linkages in QNM10-50. Providing a rationale grounded in prior work, or at least show a sensitivity check to validate that 10% increments are informative.

Although we agree that a sensitivity might be useful to better understand the specific level to which the linkage strength would impact the overall model, the attempt here was to use a systematic reduction of link strengths rather than to examine thresholds or breakdown points of the model. As we used estimates of diet and mortality from the original Rpath model (which have varying levels of estimation defined in the pedigree table) to determine the linkage strengths, we wanted to consider the practicality of how to use this information to build qualitative models without an existing quantitative ecosystem model. Thus, we attempted to classify major vs moderate vs weak interactions at reasonable increments that would be feasible to estimate interactions under data depauperate scenarios. We determined that 6 qualitative models seemed like a balanced number to examine in this study. Removing linkages above 50% interaction would result in some lost model elements (functional groups) that would no longer be linked. We have made some adjustments in the text at lines 162-166 to clarify these points.

Defining a result as positive/negative if ≥600 out of 1000 simulations show consistent direction is a bit arbitrary. I would suggest explaining this as a “majority rule” threshold or provide statistical reasoning (e.g., 95% confidence threshold would be 975+ under binomial assumptions).

Thank you for this advice. We have included in the text that this was a “majority rule” threshold, as we wanted to depict 50/50 confidently. A difference of 6 runs (e.g. 506) did not seem indicative of a true change, but within 100 (e.g. 600) we felt more confident about the change. This was agreed by consensus by the authors as subject matter experts. We have also included more examples of published work that make similar delineations without using a statistical threshold. Changes were made at lines 227-229.

The interpretation of mixed vs. neutral needs more rigor. These are currently treated as categorical, yet they reflect different aspects: low effect size vs. high variance. I would suggest considering reporting proportion of runs with positive/negative results for all nodes to clarify this distinction.

The results in Figure 3 do show the scale of the results for each model element from each perturbation (size of the points are based on the number of simulation results out of 1000) as we wanted to be transparent about these results. We determined that neutral responses were no change for the node, while mixed nearly equal positive and negative results. We have made these results publicly available in the github that we have now included as part of the supplements.

In the text we have made it clearer that these results are reflecting different aspects of not reacting (neutral) vs reacting in different directions (mixed) to reflect the low effects sizes vs high variances, respectively in lines 326-374.

### Response to Reviewer #2

Thank you for your thoughtful review. We appreciate your attention to detail in providing the comments in line. In the text, we have resolved typos, but respond to your in-line comments below:

- Line 131: Link complexity are the same from Rpath to QNM0 Figure 2. Main differences between the base model and the qualitative model is one has link strength, one retains just the direction (positive or negative) as shown in Figure 1. We have adjusted the figure caption to reflect these points.
- Line 153: The values are for individual linkages so representing the relationship between one model element to another model element which represents linkages either as prey and/or predator.
- Line 186-187: We cannot sum the biomass of the fishery as is done for other model elements because fisheries don’t have biomass. We summarized the fishery catch in this way so that it is similarly treated as other functional groups (model elements).
- Line 190: Yes there was no perturbation applied to the first 10 years of the Ecosense runs.
- Line 235: changed to “out of” (typo!)
- Line 318: We realized we were inconsistent here to the previous paragraph where we presented similar results. To remain consistent and avoid confusion, we included “or” in the bracket. This should reflect that the results are mirrored, we discuss positive (or negative). This is to keep the results more concise while remaining accurate.
- Lines 440-448: we removed some of the results details in this section of the discussion to avoid being overly repetitive.
- Line 478: Although fishery is treated here similarly to a functional group, we did not notice much difference in the way that perturbations to the fishery impacted the models compared to other perturbations. We wanted to note here that components with similar linkage structure to seals would not be well examined with the models used in this study.
- Line 491: Yes. We would consider an ensemble model as a possibility, but here considering data depauperate scenarios we thought suites of models might be more appropriate.
- Line 475: Included suggested reference
